# Identification and characterization of a novel β-glucosidase via metagenomic analysis of *Bursaphelenchus xylophilus* and its microbial flora

**DOI:** 10.1038/s41598-017-14073-w

**Published:** 2017-11-01

**Authors:** Lin Zhang, Qiang Fu, Wenpeng Li, Bowen Wang, Xiaoyan Yin, Suyao Liu, Zhaonan Xu, Qiuhong Niu

**Affiliations:** 1 Department of Life Science and Biotechnology, Nanyang Normal University, Nanyang, 473000 P.R. China; 2 State Key Laboratory of Cotton Biology, Henan Key Laboratory of Plant Stress Biology, School of Life Sciences, Henan University, Kaifeng, 475004 China

## Abstract

β-glucosidases catalyze the final step of cellulose hydrolysis and are essential in cellulose degradation. A β-glucosidase gene, *cen502*, was identified and isolated from a metagenomic library from *Bursaphelenchus xylophilus* via functional screening. Analyses indicated that *cen502* encodes a 465 amino acid polypeptide that contains a catalytic domain belonging to the glycoside hydrolase family 1 (GH1). Cen502 was heterologously expressed, purified, and biochemically characterized. Recombinant Cen502 displayed optimum enzymatic activity at pH 8.0 and 38 °C. The enzyme had highest specific activity to p-nitrophenyl-β-D-glucopyranoside (pNPG; 180.3 U/mg) and had *K*_*m*_ and *V*_*max*_ values of 2.334 mol/ml and 9.017 μmol/min/mg, respectively. The addition of Fe^2+^ and Mn^2+^ significantly increased Cen502 β-glucosidase activity by 60% and 50%, respectively, while 10% and 25% loss of β-glucosidase activity was induced by addition of Pb^2+^ and K^+^, respectively. Cen502 exhibited activity against a broad array of substrates, including cellobiose, lactose, salicin, lichenan, laminarin, and sophorose. However, Cen502 displayed a preference for the hydrolysis of β-1,4 glycosidic bonds rather than β-1,3, β-1,6, or β-1,2 bonds. Our results indicate that Cen502 is a novel β-glucosidase derived from bacteria associated with *B. xylophilus* and may represent a promising target to enhance the efficiency of cellulose bio-degradation in industrial applications.

## Introduction

Cellulose is the most abundant polysaccharide in nature and is the primary component of plant cell walls^1^. Moreover, it is the most abundant renewable carbon source available. Consequently, it is attractive for the production of different chemical compounds and biofuels^2^. However, considerable agricultural, industrial, and municipal cellulosic wastes have accumulated due to the lack of appropriate treatment, which represents an environmental pollution risk^3^. Efficient degradation and hydrolysis of cellulose requires the synergistic activities of three types of enzymes: endoglucanases (EGs), cellobiohydrolases (CBHs), and β-glucosidases (BGs)^4^. EGs and CBHs degrade native cellulose synergistically, generating cellobiose, which is a product inhibitor for these enzymes.

β-glucosidases (EC 3.2.1.21), which are widely distributed naturally, cleave the disaccharide cellobiose into two molecules of glucose that represent the end products of cellulose hydrolysis^5–13^. β-glucosidase activity is considered the rate-limiting factor in cellulose degradation by eliminating cellobiose inhibition on EGs and CBHs, thus allowing more efficient cellulolytic enzyme function. Therefore, the supplementation of this enzyme to cellulose preparations has been recommended to obtain higher degradation rates and cellulose saccharification activity^6^. However, efficient cellulose hydrolysis requires a physiologically stable β-glucosidase in order to utilize abundant cellulosic waste material on an industrial scale^7^. Recently, the identification of novel β-glucosidases from extreme and unique environments has become an attractive strategy to identify potential industrial targets.

Currently, most of the β-glucosidases used in industrial applications are derived from fungal species including *Trichoderma reesei* ^8^, *Phanerochaete chrysosporium* ^9^, *Aspergillus terreus* ^10^, *A. niger* ^11^, *A*. *oryzae* ^12^ and *A. fumigatus* ^13^. However, a number of little-studied, non-fungal isoforms may provide novel and useful targets for industrial applications. For example, there has been little investigation of β-glucosidases from the pinewood nematode (PWN), *Bursaphelenchus xylophilus* (Bx), which feeds on plant cells of trees. Bx feeding leads to the disruption of pine tissue and results in lethal wilt. The nematodes use needle-like feeding structures, termed stylets, that pierce cell walls and ingest nutrients from the cytoplasm^14^. The proteins secreted from the stylet likely consist of cell-wall degrading enzymes that mediate the interaction between nematodes and host plants. Consequently, we hypothesized that Bx and its associated microbes represent a potentially ideal and valuable resource for obtaining industrially useful β-glucosidases.

PWN Bx is an important and invasive parasitic nematode that has caused heavy tree mortality in introduced regions, including Japan, Korea, China, and Portugal^15^. Bx nematodes are characterized by rapid reproduction, which contributes to tree death^16^. Recently, Bx-associated bacteria were found to enhance Bx pathogenicity. For example, Oku *et al*. isolated epiphytic bacteria from Bx that produce toxins that cause pine wilt disease (PWD)^16^. Further, phytotoxins produced by Bx-associated bacteria cause PWD-like symptoms in pine seedlings^17^. Wu *et al*. observed endophytic bacteria in PWN using transmission electron microscopy and then successfully isolated the bacterial species *Stenotrophomonas maltophilia* from Bx. They concluded that endophytic bacteria exist widely in Bx that dwell in different pine species and areas. Moreover, they suggested that Bx strains that exhibit differing virulence possessed differing endophytic bacteria. The endophytes exhibit correspondingly diverse carbon metabolisms, which suggests that certain endophytic species and carbon metabolisms might be related to Bx virulence^18^. These results, taken together, indicate that Bx nematodes harbor a diversity of hydrolysis enzymes including cellulase, hemicellulase, ligninolytic enzymes, and β-glucosidases that aid in the synergistic degradation of plant wall structures.

Here, we constructed a metagenomic library for Bx and its associated microbial communities in order to screen for β-glucosidase genes that could be used industrially. One gene, *cen502*, that encodes a β-glucosidase was detected, cloned, sequenced, and expressed in *Escherichia coli* BL21. Cen502 enzymes were then purified and characterized. The novel β-glucosidase reported here has potential application in glycobiotechnology, and we further propose a biological role for the characterized enzyme in the degradation of cellulose.

## Results

### Construction and screening of a metagenomic library

**Figure 1:**
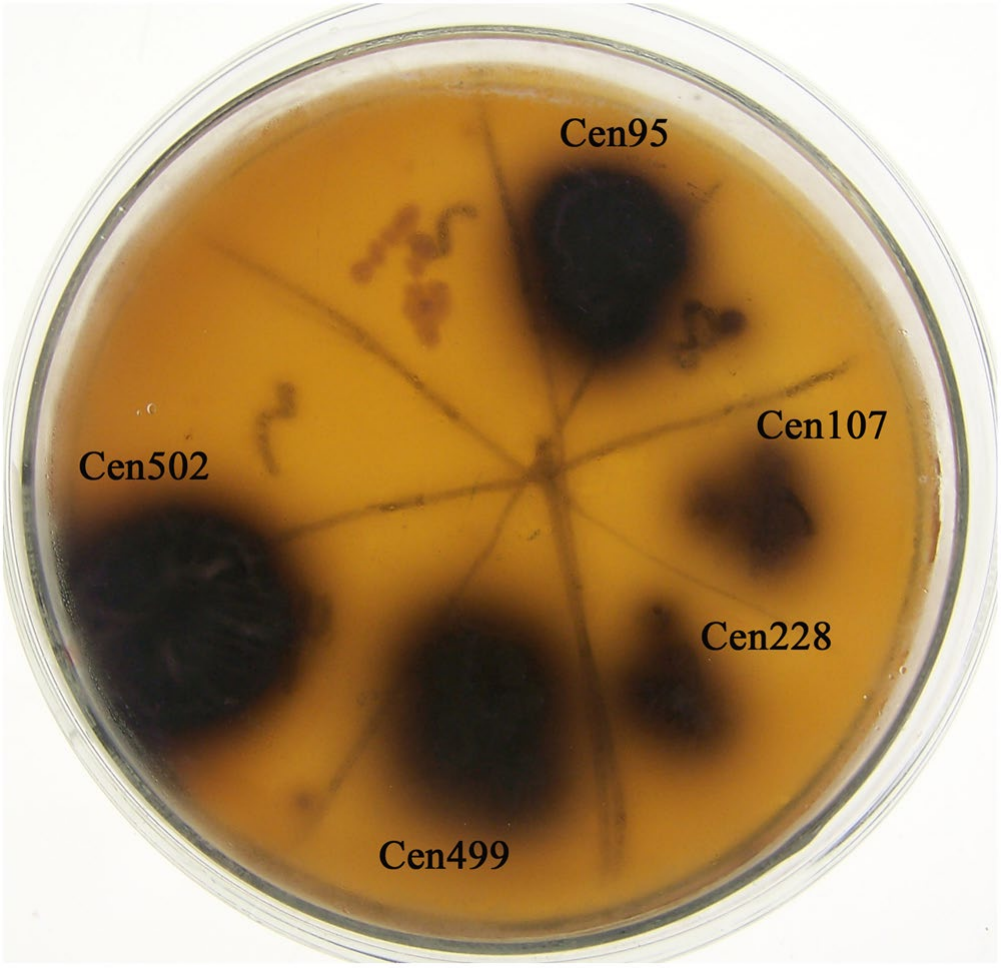
Screening of β-glucosidase activity in the metagenomic library on screening plates. Five clones expressing relatively strong β-glucosidase activity were obtained: Cen95, Cen107, Cen228, Cen499, and Cen502. Arrows indicate clone names. Clone Cen502 displayed the strongest activity and was selected for further research.

A metagenomic library comprising 4,699 clones was constructed from total community DNA that was isolated from Bx and its associated microbial communities. Restriction endonuclease analyses suggested that clone inserts ranged from 1.5 to 5.5 kbp, with an average size of ~3.0 kbp. The inserts also exhibited distinct restriction patterns. Clones were transferred to screening plates to assess β-glucosidase activity. In total, five clones (Cen95, Cen107, Cen228, Cen499, Cen502) that expressed β-glucosidase activity were isolated. A single clone, Cen502, was obtained that expressed relatively strong β-glucosidase activity, as indicated by the production of the largest black halo in β-glucosidase screening (Fig. 1). Cen502 was therefore selected for further analyses.

### Sequence and structural analysis of the β-glucosidase gene *cen502*

The Cen502 insert DNA length was 2,670 bp. BLAST analysis revealed the presence of an open reading frame (ORF) consisting of 1,398 bp. The ORF comprised a full-length β-glucosidase gene (*cen502*) that encoded a protein consisting of 465 amino acids. The gene sequence for *cen502* was submitted to GenBank (accession KX095398, data not released) and a national invention patent has been successfully acquired for the gene (patent No. 201410014799.X).

**Figure 2:**
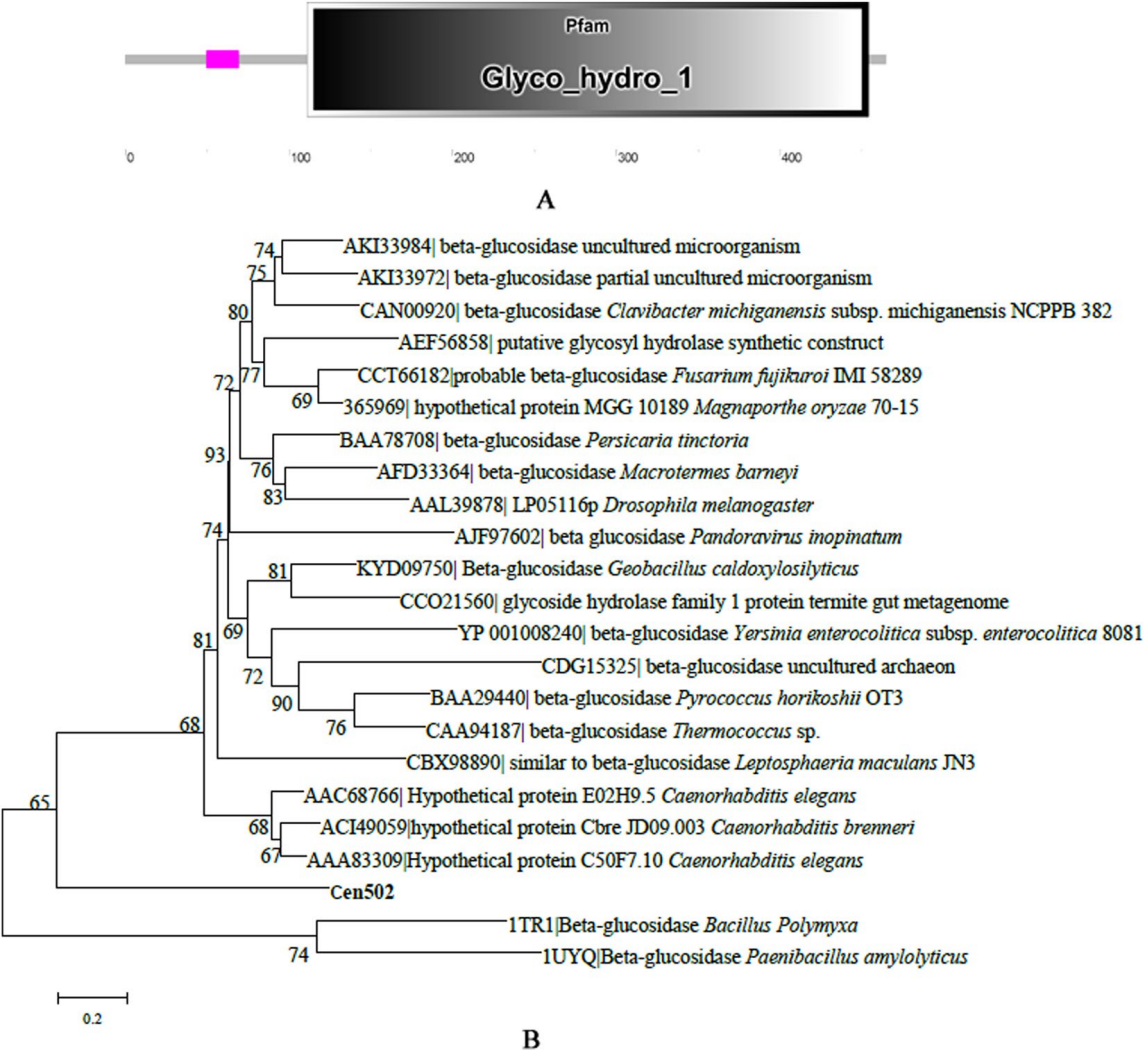
Analysis of the Cen502 amino acid sequence. (**A**) Predicted modular architecture of Cen502. (**B**) Neighbor-joining phylogenetic tree based on 27 β-glucosidase sequences from various organisms. The analysis depicts the evolutionary relationship between Cen502 and related proteins. Bootstrap values of 60% or above are shown at the nodes. Scale bar corresponds to a genetic distance of 0.2 substitutions/site.

Based on calculations using the ExPASy program http://cn.expasy.org/tools/pi_tool.html, the theoretical molecular mass of Cen502 is 52.8 kDa and the pI is 8.7. Sequence analysis with the SMART software package indicated that the putative protein had one glycosyl hydrolase family 1 domain (extending from amino acid positions 111 to 454; Fig. 2A). Conserved domain searches in NCBI confirmed the results from the SMART analysis, indicating that Cen502 represents a probable GH1 β-glucosidase. The protein shared 62% amino acid identity with β-glucosidase A from *Bacillus polymyxa* and 47% amino acid identity with the β-glucosidase from *Paenibacillus amylolyticus*.

Phylogenetic analyses were then conducted to place Cen502 among known GH1 family and β-glucosidase diversity (Fig. 2B). Twenty-two β-glucosidase proteins from various organisms were selected from the CAZY database for the phylogenetic analysis. The dataset included five β-glucosidase proteins from bacteria, three from fungi, three from nematodes, four from other Eukaryota, three from Archaea, one from a virus, and three from unclassified organisms. Comparative analyses indicated that Cen502 exhibited between 47% and 62% sequence identities to homologous bacterial proteins (e.g. *B. polymyxa*), and between 19% and 22% sequence identities to homologous nematode proteins (e.g. *C. elegans*). However, Cen502 was not closely related to other β-glucosidase family proteins, suggesting that it is a potentially novel isoform.

Multiple sequence alignment analysis with other β-glucosidases indicated that Cen502 is a typical GH1 β-glucosidase (Supplemental Fig. 1). The Cen502 residues E161 and E369 corresponded to the conserved glutamic acid residues that are involved in the catalytic activity of GH1 enzymes. These results are consistent with the functional domain predictions and a putative active site in the cold-active β-glucosidase BglMKg^19^. Moreover, the residues D244, R340, K351, and H388 are hypothesized to form hydrogen bonds with the substrate sugar hydroxyls.

**Figure 3:**
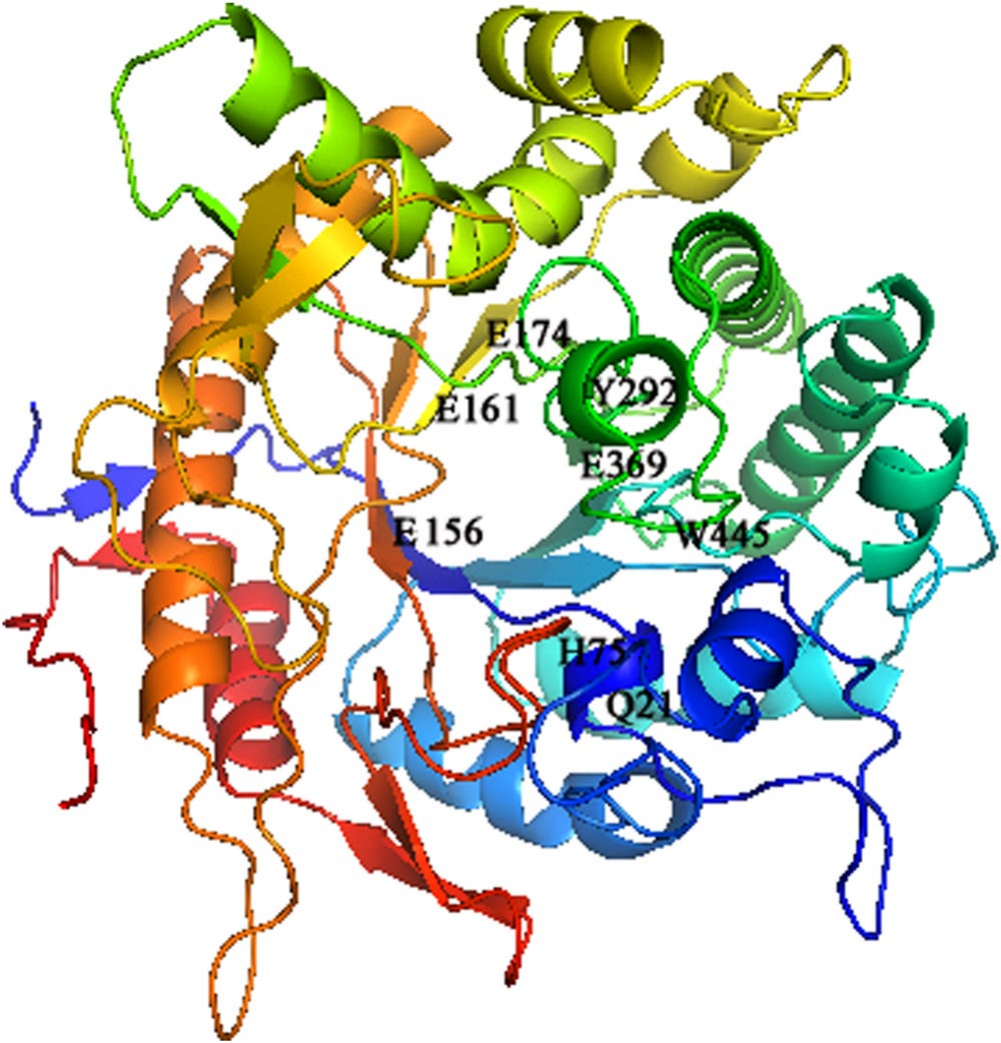
Structural model of the β-glucosidase Cen502.

The Cen502 structure consisted of an eightfold β/α architecture that is typical for family 1 glycoside hydrolases (Fig. 3). The glutamic acid residues E156, E161, and E174 were the predicted active sites among the conserved amino acids. The residues Q21, H75, Y292, E369 and W445 were also predicted to be conserved, and form the typical pocket-shaped substrate recognition domain^20^. Consequently, these residues determine substrate recognition. The structure features a wide and extended cavity surrounding the active-center cavity, which seems to coincide with the accommodation of a wide range of substrates.

### Expression and purification of Cen502

**Figure 4:**
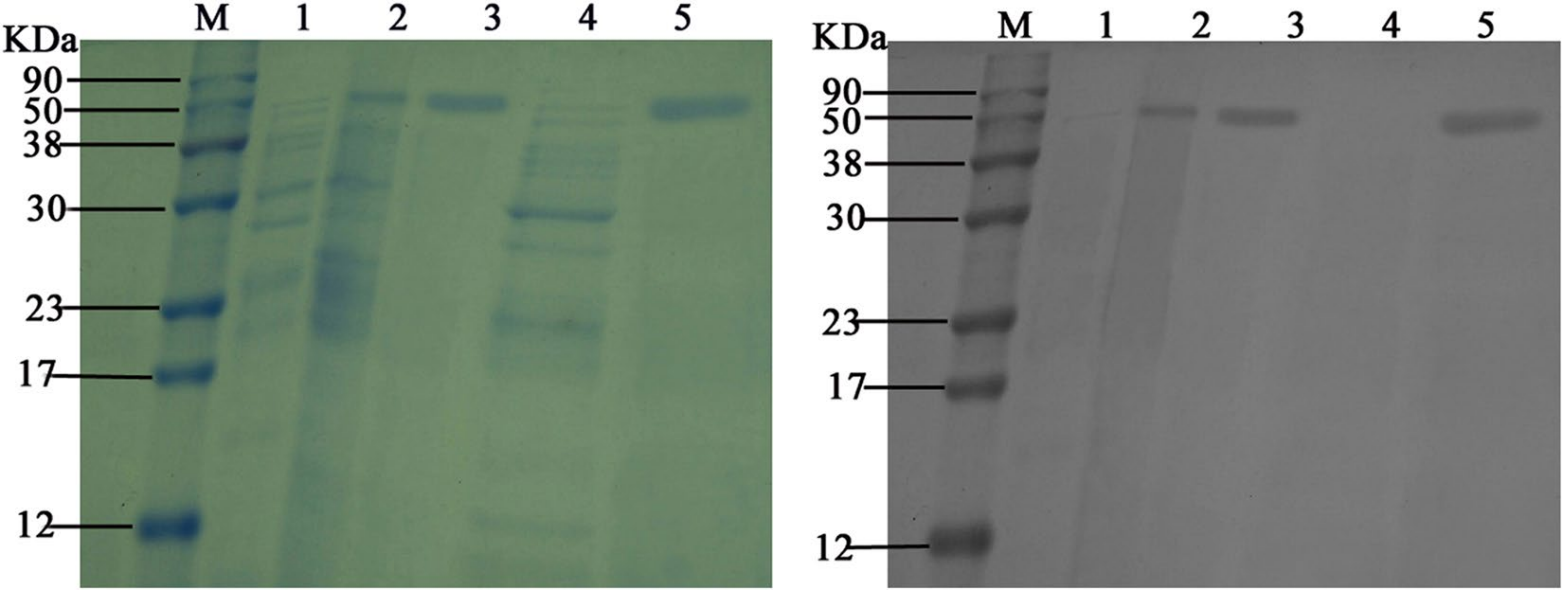
(**A**) SDS-PAGE analysis of the purified, recombinant Cen502 protein. M, marker proteins; lane 1, IPTG-uninduced crude extract of BL21 (DE3) carrying pET-Cen502; lane 2, IPTG-induced crude extract of BL21 (DE3) carrying pET-Cen502; lane 3, eluted Cen502 protein after purification; lane 4, crude extract of BL21 (DE3) carrying pET30a (+) vector; lane 5, desalted Cen502 protein. (**B**) Characterization of Cen502 by Western blot. M, marker proteins; Lanes 1–5 are the same as in A.

In order to characterize the biochemical properties of Cen502, it was heterologously expressed as an N-terminal His-tag fusion protein using the pET-30a (t) expression system under the control of the T7 lac promoter in *E. coli* BL21. β-glucosidase activity was detected after cells were harvested and disrupted by sonication, suggesting that recombinant Cen502 was secreted and expressed in a soluble form. SDS-PAGE analysis of the fusion enzyme before and after purification, in addition to Western blot analysis after dialysis, are provided in Fig. 4 and Supplemental Fig. 2. The purity of the resulting elution was confirmed by the presence of a single band after staining on a 12% SDS-PAGE gel (Fig. 4A). The molecular mass of the purified enzyme was estimated at approximately 52 kDa by SDS-PAGE, which is consistent with the predicted MW value. Western blot analysis yielded a 52-kDa protein band for the IPTG-induced crude extract of BL21 (DE3) carrying pET-Cen502, eluted Cen502 protein after purification, and desalted Cen502 protein (Fig. 4B, lanes 2, 3, and 5, respectively). No hybridization signal was detected for the negative controls including IPTG-uninduced crude extract of BL21 (DE3) carrying pET-Cen502, and crude extract of BL21 (DE3) carrying the pET30a (+) vector (Fig. 4B, lanes 1 and 4, respectively).

### Characterization of Cen502

**Figure 5:**
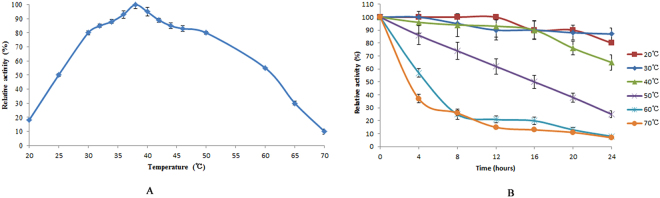
Effect of temperature on the activity and stability of Cen502. (**A**) Effect of temperature on Cen502 activity; (**B**) Effect of temperature on Cen502 stability.

**Figure 6:**
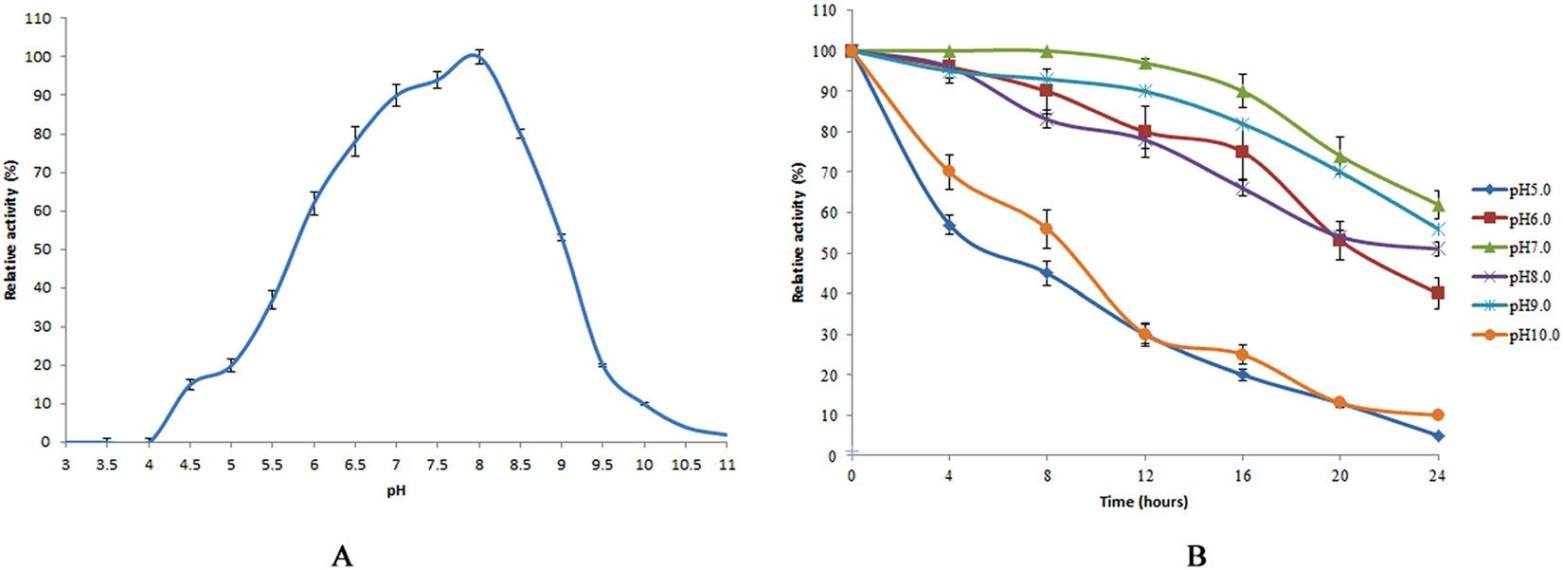
Effect of pH on the activity and stability of Cen502. (**A**) Effect of pH on Cen502 activity; (**B**) Effect of pH on Cen502 stability.

Temperature-dependence studies indicated that the optimal temperature of Cen502 was 38 °C. Only 25% and 8% of the original Cen502 activity was recovered when analyzed at 50 °C and 60 °C, respectively, for 24 h, which indicated weak thermal stability (Fig. 5). pH optimization studies at 38 °C indicated that purified Cen502 had maximum hydrolytic activity at pH 8.0 (Fig. 6). The enzyme showed comparatively good pH stability by retaining 97% and 90% of its activity at pH 7.0 and pH 9.0, respectively, for 12 hrs. After 24 hrs of incubation, the relative activity of Cen502 was 62%, 51%, and 56% at pH 7.0, 8.0, and 9.0, respectively.

**Figure 7:**
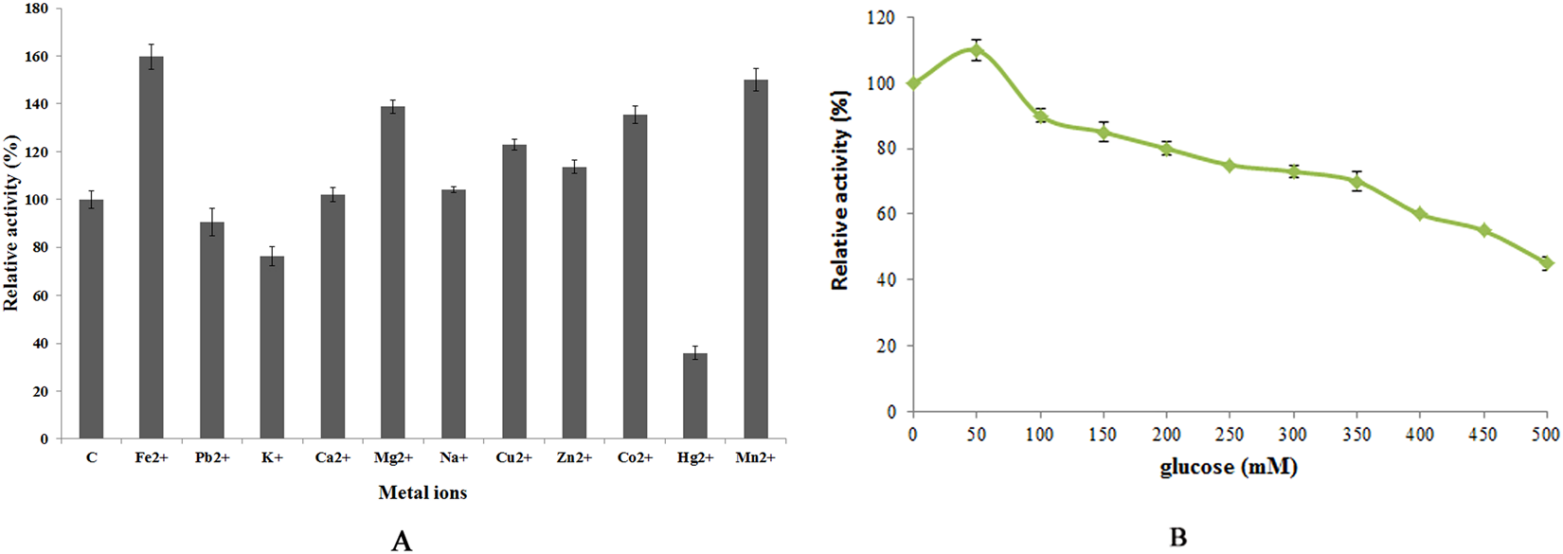
Effects of metal ions on Cen502 activity.

The effects of metal ions on Cen502 activity are shown in Fig. 7A. The addition of 0.05 mol/L Fe^2+^ and Mn^2+^ enhanced β-glucosidase activity by ~60% and 50%, respectively. Mg^2+^, Zn^2+^, and Cu^2+^ also enhanced Cen502 activity by 14–40%. In contrast, Pb^2+^ and K^+^ addition decreased β-glucosidase activity by 10% and 25%, respectively. Na^+^ had little effect on the activity of Cen502.

Substrate specificity of Cen502 was determined under optimal conditions after addition of various polysaccharides at 1% concentrations (Table 1). Cen502 was maximally active against pNPG, with a specific activity of 101.7 ± 5.2 U/mg. Cellobiose (β-1,4-linked) and lactose (β-1,4-linked) were the most preferred substrates, followed by salicin (β-linked), lichenan (β-1,3, β-1,4-linked), laminarin (β-1,3, β-1,6-linked), and sophorose (β-1,2-linked). Purified enzymes had very little to no activity towards carboxymethyl cellulose (CMC), xylan, or avicel substrates. Taken together, the results indicated that Cen502 had the highest activities toward β-1,4-linked saccharides. Further, the enzyme was found to be efficient in the hydrolysis of β-1,3, β-1,6, and β-1,2-linked polysaccharides. Thus, Cen502 may have potential application in diverse saccharide degradation processes that are important in various biotechnological fields.

**Table 1:** Cen502 hydrolysis activity on various polysaccharide substrates.

Substrate	Specific activity^a^ (U/mg) ± SD	Relative activity (%)
pNPG	180.3 ± 9.2	100.0
Sophorose (β-1,2-linked)	24.0 ± 2.2	13.3
Laminarin (β-1,3, β-1,6-linked)	29.7 ± 2.0	16.5
Salicin (β-linked)	73.6 ± 4.1	40.8
Lichenan (β-1,3, β-1,4-linked)	49.9 ± 3.8	27.7
Lactose (β-1,4-linked)	126 ± 6.4	69.9
Cellobiose (β-1,4-linked)	127.8 ± 6.6	70.9

^a^Ten min. incubation times were used for activity measurements, and they were performed in triplicate to assess standard deviation.

The activity of Cen502 towards pNPG was enhanced by glucose at concentrations lower than 50 mM (Fig. 7B). In the presence of 50 mM glucose, the activity of Cen502 increased to a maximum value of 10% more than that of the control without glucose. Enzyme activity was gradually inhibited with increasing glucose concentrations.

**Figure 8:**
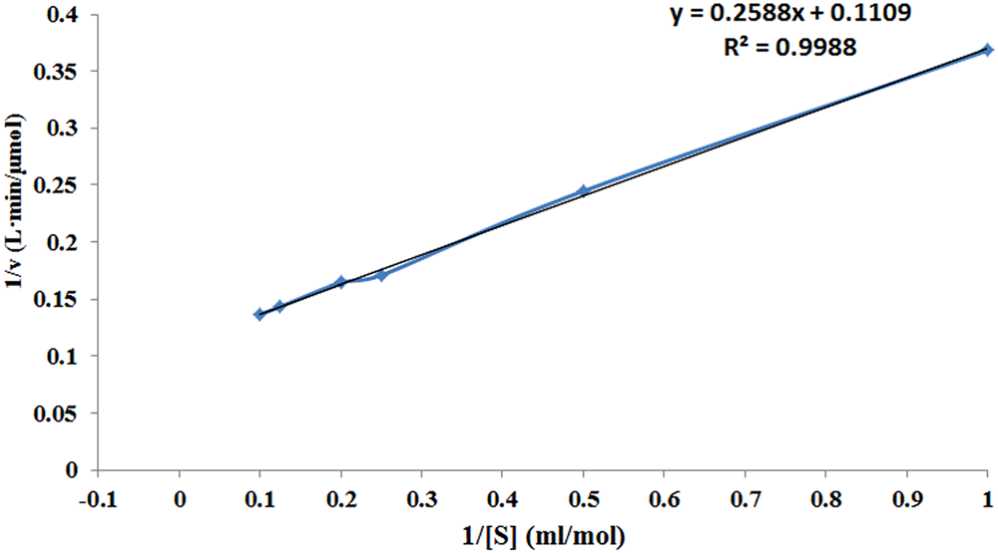
Kinetic constants determined for Cen502 activity towards pNPG using the Lineweaver-Burk assay.

The enzymatic kinetics (substrate affinity and maximum rate) of Cen502 were then calculated to further characterize its activity. Based on the equation 1/ѵ = *K*_*m*_·1/*V*_*max*_·1/[s] + *V*_*max*_, a straight line was obtained by drawing 1/ѵ ~ 1/[s]. The intercepts of the x-axis and y-axis are then −1/*K*_*m*_ and the 1/*V*_*max*_, respectively, allowing the calculation of a maximum rate (*V*_*max*_) and substrate affinity (*K*_*m*_). Using these calculations, the *K*_*m*_ and *V*_*max*_ of recombinant Cen502 towards pNPG was 2.334 mol/ml and 9.017 μmol/min, respectively (Fig. 8).

## Discussion

Lignocellulosic biomass is one of the largest renewable resource reserves on Earth. With intensifying fossil fuel shortages and increasing environmental pollution, effective use of these low-cost resources to produce biofuel and biochemical products is an efficient way to aid in efforts to relieve the current energy crisis, protect environments, and maintain sustainable economic development. Lignocellulose degradation metabolic pathways are naturally abundant, and conversion by enzymes is one of the most efficient pathways of lignocellulosic degradation^21^. Consequently, urgent demand for lignocellulose degradation into its component sugars has led to increased investigation of novel cellulases from extreme and unique environments.

Here, we identified a novel β-glucosidase gene from the microbial communities of *B. xylophilus*, Bx, by constructing a metagenomic library comprising 4,699 clones with DNA fragment inserts. From this metagenomic library, five β-glucosidase-positive clones were obtained (Fig. 1). Physiological screening of the cloned inserts indicated that clone Cen502 displayed maximal β-glucosidase activity. Hydrolysis of pNPG was used to assess β-glucosidase activity, and Cen502 was confirmed as having the highest β-glucosidase activity of the five isoforms detected here. Moreover, the Cen502 clone insert was identified as a β-glucosidase gene via nucleotide and amino acid sequence analysis.

The unique properties of Cen502 are summarized as follows:

1. Cen502 is characterized by high catalytic efficiency at high pH, as indicated by its optimum enzymatic activity at pH 8.0. The optimum pH of purified Cen502 was higher than most known β-glucosidases, which typically exhibit optimal pHs below 7.0^13,15,22–25^. Of note, Cen502 is active over a wide range of pHs, retaining 56% of its activity at pH 9.0, and is stable over the pH range 5.0–9.0. Although the β-glucosidase production of many bacteria, archaea, and fungi has been studied, the majority of their pH optima are acidic. For example, the thermostable β-glucosidase from *A. fumigatus* Z5 has optimal activity at pH 6.0 and is stable over the pH range 4–7^13^. Further, the purified β-glucosidase from the digestive fluid of palm weevil larvae showed maximal activity at pH 5.0 and was stable in the pH range 5.0–6.0^23^. The β-glucosidase from *Fusarium oxysporum* has optimal activity at pH 5.0, and the enzyme is stable over the pH range 4.0–7.0^24^. Likewise, the optimal pH of the β-glucosidase Unglu135B12 from cattle rumen is 5.0, and the enzyme is stable only in the pH range 5.0–6.0^25^. The β-glucosidase *Ho*BGLA from *Halothermothrix orenii* has maximal activity at pH 6.0 and is stable in the pH range 4.5–7.5^26^. Lastly, the thermally stable β-glucosidase from *Pyrococcus furiosus* exhibits an optimum activity level at pH 6.0 and is nearly inactivated at extreme pHs (pHs 4.0 and 9.0)^27^.
  β-glucosidases have great application prospects in many industrial fields, including medicine, textiles, chemical engineering, paper engineering, food and fermentation industries, commercial detergent manufacturing, tobacco processing, wastewater treatment, and in feed additives. Some chemical processes in these fields are carried out under strongly alkaline conditions. Therefore, the identification of alkaline-active β-glucosidases is particularly promising for application in the above settings. Thus, the alkaline-active Cen502 may be a promising candidate for lignocellulose saccharification in industrial applications.
2. The molecular mass of the β-glucosidase protein Cen502, 52 kDa, is smaller than previously identified β-glucosidases. For example, the molecular mass of the β-glucosidase Unglu135B12 from cattle rumen is ~85 kDa^25^, while the β-glucosidase from *A. niger* KCCM 11239 can reach 123 kDa^17^, which is the largest size known for this group of enzymes.
3. Cen502 was unstable above 40 °C. The optimal temperature of 38 °C for Cen502 was the same as that recorded for a β-glucosidase from cattle rumen^25^ and is similar to the optimal temperature of the β-glucosidase from *Proteus mirabilis* VIT117 that was grown on prawn shells^28^. However, higher temperature optima are known for β-glucosidases: from the digestive fluid of palm weevil larvae (55 °C)^23^, *A. fumigatus* Z5 (60 °C)^13^, *A. niger* KCCM 11239 (70 °C)^22^, and *H. orenii* (65–70 °C)^26^.
4. The specific activity of Cen502 against pNPG was measured as 180.3 U/mg at pH 8.0 when assayed at 38 °C.
5. Addition of Fe^2+^ and Mn^2+^ could enhance Cen502 β-glucosidase activity up to 160% and 150%, respectively, compared with assays without the addition of metals.
6. Cen502 hydrolyzes a diversity of saccharide substrates, including cellobiose, lactose, salicin, lichenan, laminarin, and sophorose. In addition, it preferentially hydrolyzes β-(1,4)-linked polysaccharides over β-(1,3)-, β-(1,6)-, and β-(1,2)-linked polysaccharides. A possible explanation for its broad substrate specificity is the wide and extended cavity structure that surrounds the active-center cavity, which was determined by homology modeling^20^.

In conclusion, a β-glucosidase family 1 glycoside hydrolase, Cen502, was identified from a metagenomic library consisting of DNA from the pinewood nematode *B. xylophilus* and its associated microbial communities. Functional screening and expression in *E. coli* permitted the isolation and characterization of this enzyme. Sequence analysis revealed that *cen502* encoded a protein comprising 465 amino acids. SDS-PAGE analysis of the purified protein suggested that Cen502 was a monomeric enzyme with a molecular mass of 52 kDa. The optimum temperature and pH for Cen502 β-glucosidase activity was 38 °C and pH 8.0, respectively, although the enzyme was stable at pHs ranging from 7.0 to 9.0. Enzyme activity was significantly enhanced by Mn^2+^ and Fe^2+^ addition but decreased after Pb^2+^ or K^+^ addition. The enzyme hydrolyzed a wide range of substrates, including cellobiose, lactose, salicin, lichenan, laminarin, and sophorose. The recombinant Cen502 showed highest specific activity towards pNPG (180.3 U/mg) under optimal conditions, and its *K*_*m*_ and *V*_*max*_ values were 2.334 mol/ml and 9.017 μmol/min/mg, respectively. Based on these results, the enhancement of Cen502 activity appears to be very tractable.

Compared with commercially available β-glucosidases, Cen502 shows better stability over a wide pH range (from pH 5.0 to 9.0). The unique properties outlined above for Cen502 conversion of cellobiose into glucose could result in its application in many industrial settings. Additionally, endo- and exocellulases can degrade synergistically during the hydrolysis of cellulose to generate cellobiose, which is a product inhibitor for cellulases^23^. Thus, β-glucosidases play important roles in scavenging the end product cellobiose by cleaving β-(1-4)-linked polysaccharides to generate glucose but are also important in the regulation of endo- and exocellulase synthesis. These β-glucosidases hydrolyze oligosaccharides to glucose immediately during the process of lignocellulose degradation, which then restricts the accumulation of inducers and the subsequent induction of cellulase genes. Thus, our discovery and characterization of the Cen502 enzyme provide a theoretical framework for unraveling the regulatory mechanisms of lignocellulolytic enzyme production.

## Materials and Methods

### Ethic Statement

No specific permission was required for the described field studies. Specific permits were also not required for sampling locations and activities. The sampling location was not privately owned or protected from research activities. All studies were performed according to the laws of the People’s Republic of China.

### Construction of a metagenomic library and screening for β-glucosidase clones

**Figure 9:**
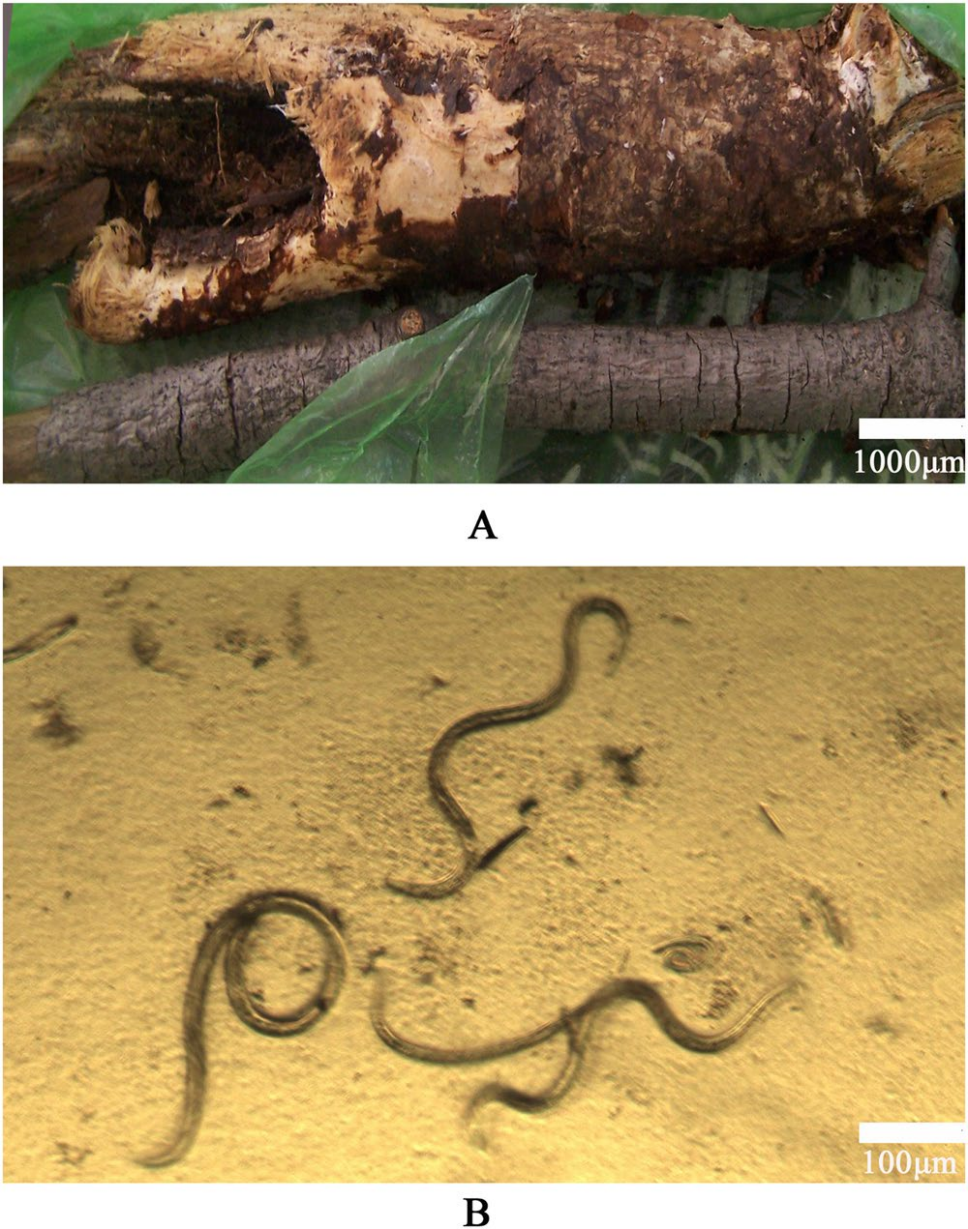
Source of samples. (**A**) *Pinus armandi Franch* samples obtained from PWD epidemic areas in the Xiaoqinling Nature Reserve located in Sanmenxia city, Henan province, China; (**B**) *B. xylophilus* nematodes found in *Pinus armandi Franch* samples.

*Pinus armandi Franch* samples were chosen from pine wilt disease (PWD) epidemic areas of the Henan Xiaoqinling Nature Reserve that is located in Sanmenxia city, Henan province, China (Fig. 9A). Nematodes were identified and isolated from the *Pinus armandi Franch* samples (Fig. 9B). Nematodes were classified by phenotypic and genotypic characteristics, and confirmed as *B. xylophilus* (Bx). Nematodes were then inoculated onto fungus *Botrytis cinerea* PDA plates and cultivated at 25 °C for 9 d. Cultured nematodes were then separated using the Baerman funnel technique^29^, and an aqueous suspension of nematodes (~50,000 worms) was prepared for use as a working stock.

DNA was extracted from Bx and their associated microbial communities using methods described in our previous analyses^30^. Samples were washed three times with sterile water and then ground with liquid nitrogen for 10 min. Ground samples were then mixed with DNA extraction buffer, proteinase K, and SDS. The mixtures were incubated in a 65 °C water bath overnight. Genomic DNA was then obtained using phenol chloroform extractions. DNA quality was evaluated using a Nanodrop spectrophotometer, where a A260/A280 ratio between 1.8 and 2.0 was used as the quality criterion. DNA fragments were recovered from gels by electroelution and then partially digested using the BamHI restriction enzyme. Digestion products were specified in the 2.5–10 kbp size range. After purification, DNA fragments were ligated into BamHI-digested pUC118, and the ligated products were transformed into *E. coli* JM109 cells. Transformed cells were plated onto LB agar plates containing esculin hydrate (0.1%), ferric ammonium citrate (0.25%), and chloramphenicol (22.5 μg/ml). Clones were then screened for β-glucosidase activity according to methods described by Eberhart *et al*.^31^. After incubation at 37 °C for 20–24 h, clones exhibiting a black halo around their colonies were selected to screen for β-glucosidase activities. Enzyme activity strength was initially qualitatively assessed by comparing the size of each black halo.

### Sequence and structure analysis of β-glucosidase gene *cen502*

The clone insert from the colonies exhibiting the highest β-glucosidase activity was sequenced using a BigDye sequencing kit on an ABI 377 DNA sequencer. The open reading frame (ORF) of the target sequence was analyzed using DNAMAN6.0 software. Analysis of the deduced amino acid sequence and ORF searches were performed using the BLAST program in NCBI. Domain architecture and conserved domains were identified with the SMART program (http://smart.embl-heidelberg.de). Phylogenetic analyses were conducted using the MEGA5.0 software with the neighbor-joining algorithm^32^. The robustness of the phylogenetic tree topology was evaluated using the bootstrapping with resampling method of Felsenstein and 1000 bootstrap replicates^33^. Sequences that were most similar to Cen502 based on amino acid BLAST searches against the NCBI and the PDB databases were chosen for inclusion in the phylogenetic analyses.

The 3D structure of Cen502 was obtained using the EasyModeller 2.0 software package and the crystal structures of β-glucosidases from *B. polymyxa* (PDB ID: 1TR1; a.a. identity: 63.84%) and *P. polymyxa* (PDB ID: 1UYQ; a.a. identity: 62%) as templates. These homologues were used because the deduced amino acid sequence of Cen502 showed the highest similarity with these two β-glucosidases. The Structural Analysis and Verification Server (SAVES; http://services.mbi.ucla.edu/SAVES/) was used to assess the stereochemical quality of the 3D models as described in our previous analyses^34^.

### Expression and purification of the recombinant β-glucosidase Cen502

*cen502* was amplified using PCR with a BamHI-linked sense primer P1 (5′-CGCGGATCCATGAAAAAGCTGTGTTTGC-3′), an XhoI-linked antisense primer P2 (5′-CCGCTCGAGTCCGAGCAATTCAAAGCT-3′), and the clone strain pUC118-cen502 as a template. PCR fragments were then digested with the restriction endonucleases BamHI and XhoI and then cloned into pET30a (+) which was digested with the same enzymes, thus resulting in the recombinant plasmid pET30a/Cen502. The recombinant plasmid was transformed into *E. coli* BL21 for expression. Expression was induced by adding 1.0 mM IPTG (Sigma) when cell density reached an A_600_ of 0.4–0.6. Cells were then incubated for an additional 3.5 h at 30 °C. Ni-NTA resin was used to purify the protein following the pET System manufacturer’s instructions. Protein preparations were then desalted by gravity flow using a PD-10 ultrafiltration column (GE Healthcare, Mississauga, ON, Canada) according to the manufacturer’s instructions. The desalted product was eluted with 20 mM citrate-phosphate buffer (pH 7.0), and analyzed using sodium dodecyl sulfate-polyacrylamide gel electrophoresis (SDS-PAGE). Protein concentrations were determined according to previously described methods^35^ and using bovine serum albumin (BSA) as a standard. Purified samples (15 μg) were transferred onto a PVDF membrane (Bio-Rad) for Western blot analysis. The PVDF membrane was then treated with anti-His Tag (6×) antibody to conduct immunoblots. The stained bands were clearly visible using a DAB staining kit. The culture supernatant of the strain with only the pET-30a vector was used as a negative control.

### Enzyme activity assays and protein content determination

β-glucosidase activity was measured by determining the hydrolysis of p-nitrophenyl-β-D-glucopyranoside (pNPG) (Sigma, USA) using the initial rate of colored reaction product accumulation as described by Korotkova *et al*.^36^. A 20 μl aliquot of the acquired enzyme was mixed with 180 μL of 5 mM pNPG substrate in 50 mM sodium citrate buffer (pH 7.0). After incubation at 50 °C for 10 min, the reaction was terminated by adding 100 μl of ice-cold 0.5 M Na_2_CO_3_. The release of p-nitrophenol (pNP) via enzymatic hydrolysis was indicated by the appearance of a yellow color and monitored with an xMark Microplate Spectrophotometer (Bio-Rad, Canada) at 405 nm. One unit (U) of β-glucosidase activity was defined as the amount of enzyme that released 1 μmol of pNP per minute from the substrate.

Protein content was measured with a BCA protein assay kit (Pierce, Rockford, IL, USA) using bovine serum albumin (BSA) as the standard. All enzyme activities are expressed as units per milligram of protein.

All experiments were performed with three replicates and repeated at least thrice.

### Characterization of Cen502

The effect of temperature on β-glucosidase activity was analyzed by measuring enzyme activity, as described above, at various temperatures (20 to 70 °C) in 50 mM citrate buffer (pH 7.0) for 10 min. The thermal stability of purified Cen502 was investigated by pre-incubating the enzyme solutions in 50 mM sodium acetate buffer (pH 5.0) for 4–24 h, with temperatures ranging from 20–70 °C. The remaining β-glucosidase activity of each treatment group was subsequently measured as described above. Relative activity was calculated as enzymatic activity at each temperature divided by the maximal activity at the optimal temperature^37^.

The optimal pH of Cen502 was determined by measuring activity toward pNPG at pH values between 3 and 11 at the optimal reaction temperature, as described above. The pH stability was assessed by incubating 100 μL of purified Cen502 at 4 °C for 4–24 h, and adjusting pH to 7, followed by β-glucosidase activity assays to assess the remaining activity, as described above.

The effects of metal ions on activity were assessed by adding 0.05 mol/L metals (Fe^2+^, Pb^2+^, K^+^, Ca^2+^, Mg^2+^, Na^+^, Cu^2+^, Zn^2+^, Co^+^, Mn^+^, and Hg^2+^) into reaction mixtures followed by β-glucosidase activity measurements.

β-glucosidase activity on various polysaccharides was determined by measuring the reduction of sugars with the dinitrosalicylic acid (DNS) method^38^. Activity was also assessed with other substrates by measuring the amount of reducing glucose that was present according to methods described by Lin *et al*.^39^.

Kinetic measurements (*V*_*max*_ and *K*_*m*_) were determined by directly fitting activity data to the Michaelis–Menten equation by nonlinear regression. Reactions were carried out under optimal conditions with pNPG concentrations ranging from 1 to 10 mol/ml.

## Electronic supplementary material

Supplementary Information
