# Supplementary material for: Identification and characterization of a novel β-glucosidase via metagenomic analysis of Bursaphelenchus xylophilus and its microbial flora
Source: Sci Rep. 2017 Nov 1;7:14850. doi: 10.1038/s41598-017-14073-w (PMC5665999; doi:10.1038/s41598-017-14073-w)
Supplement: Supplementary file 1 — Supplementary Information [file 41598_2017_14073_MOESM1_ESM.pdf]

Supplements

Identification and characterization of a novel β-glucosidase via metagenomic analysis of *Bursaphelenchus xylophilus* and its microbial flora

Running Head: Novel β-glucosidase isolated from *Bursaphelenchus xylophilus* and its catalytic properties

Lin Zhang[1, 2], Qiang Fu[1], Wenpeng Li[1], Bowen Wang[1], Xiaoyan Yin[1], Suyao Liu[1], Zhaonan Xu[1], Qiuhong Niu[1]*

[1]Department of Life Science and Biotechnology, Nanyang Normal University, Nanyang, 473000, P.R.China
[2]State Key Laboratory of Cotton Biology, Henan Key Laboratory of Plant Stress Biology, School of Life Sciences, Henan University, Kaifeng 475004, China

Lin Zhang and Qiang Fu contributed equally to the work.
*Corresponding author: Qiuhong Niu
Telephone: +86-377-63525086
Fax: +86-377-63525086
E-mail: qiuhongniu@hotmail.com

Supplemental figure 1:

Figure 1 legend:

Multiple alignment of the deduced amino acid sequence of Cen 502 with other bacterial counterparts from *Bacillus Polymyxa* (Bp, 1TR1), Bacteroides salanitronisand (Bs, ADY36013), and Paenibacillus amylolyticus (Pa, 1UYQ).

Supplemental figure 2:

Figure 2 legend:

The original SDS-PAGE analysis of the purified recombinant Cen502. M, marker proteins; lane 1, IPTG-uninduced crude extract of BL21 (DE3) carrying pET-Cen502; lane 2, IPTG-induced crude extract of BL21 (DE3) carrying pET-Cen502; lane 3, eluted Cen502 protein after purification; lane 4, crude extract of BL21 (DE3) carrying pET30a (+) vector; lanes 5&6, desalted Cen502 protein; lanes 7&8,blank.

```
502.seq        ..........MKKLCLLAGVYMWGTATAALQIEGAKKLCL    30
Bp.txt         ............TIFQFPQDFMWGTATAAYQIEGAYQ...    25
Bs.txt         MDKMTLREKLGQLNLPAGGDLTTGTVQNSNLAEMIRKQ..    78
Pa.txt         ............TIFQFPQDFMWGTATAAYQIEGAYQ...    25
Consensus                             gt      e

502.seq        LVPGGWKTVLDLGDVCPKACVCVQRRQRQCSLRQLSSLPS    70
Bp.txt         .EDGRGLSIWDTFAHTPGKVFNGDNGNVACDSYHRYEEDI    64
Bs.txt         .ELGGFFNVMSVEKIRELQRIAVEETRLGIPLVVGADVIH   117
Pa.txt         .EDGRGLSIWDTFAHTPGKVFNGDNGNVACDSYHRYEEDI    64
Consensus         g

502.seq        RYGVHLPTRLSYISFRG..LLATYIPTWCTWSQSRGIGRI   108
Bp.txt         RLMKELGIRTYRFSVS....WPRIFPNGDGEVNQKGLDYY   100
Bs.txt         GYQTIFPIPLALACSWDTLATERMAQISAKEATANGIAWT   157
Pa.txt         RLMKELGIRTYRFSVS....WPRIFPNGDGEVNQEGLDYY   100
Consensus                                         g

502.seq        IIVKLICLND.........NIGLPFS.TLYDRVLPQPLQL   138
Bp.txt         HRV.VDLLND.........NGIEPFC.TLYHWDLPQALQD   129
Bs.txt         FSPMVDICRDARWGRIAEGNGEDPFIGAMMARAYVRGYQG   197
Pa.txt         HRV.VDLLND.........NGIEPFC.TLYHWDLPQALQD   129
Consensus               d         n   pf             q

502.seq        AVGWGNPPRIQAFVQFAETMFREFHGKIQHWHSFNEPWCT   178
Bp.txt         AGGWGNRRTIQAFVQFAETMFREFHGKIQHWLTFNEPWC.   168
Bs.txt         ENLHESDSSMMACLKHFALYGASESGRDYNRTDMSRVQML   237
Pa.txt         AGGWGNRRTIQAFVQFAETMFREFHGKIQHWLTFNEPWC.   168
Consensus                 a             g

502.seq        IAFVIQYVNGSCPG.SDLSPDCDCCR..............   203
Bp.txt         IAFLSNMLGVHAPGLTNLQTAIDVG...............   193
Bs.txt         NEYLPPYQAAVKAGVGTVMTSFNTINGVPATADKWLIDDV   277
Pa.txt         IAFLSNMLGVHAPGLTNLQTAIDVG...............   193
Consensus                   g

502.seq        .TSSWLAHGLSVRRFRVLGTSGDSGIAPNVSWAVPYWHSE   242
Bp.txt         .HHLLVAHGLSVRRFRELGTSGQIGIAPNVSWAVPYSTSE   232
Bs.txt         LRKQWGFKGMIVTDYNSIAEMEIHGVAPLKEAGIMAMNAG   317
Pa.txt         .HHLLVAHGLSVRRFRELGTSGQIGIAPNVSWAVPYSTSE   232
Consensus              g  v            g ap

502.seq        VDKAACAR............................TISL   254
Bp.txt         EDKAACAR............................TISL   244
Bs.txt         TDMDMVAQSFLNPMEEAVNEGKVSKARIDEACRRVLEMKY   357
Pa.txt         EDKAACAR............................TISL   244
Consensus       d    a

502.seq        HSGLFLQPIFQGTIPQFLVDWFALQGGTVP..........   284
Bp.txt         HSDWFLQPIYQGSYPQFLVDWFAEQGATVP..........   274
Bs.txt         KLGLFENPYKYCNVERAQTETYSEANRAEARRIAAETFVL   397
Pa.txt         HSDWFLQPIYQGSYPQFLVDWFAEQGATVP..........   274
Consensus          f  p

502.seq        ..........IQDGDMEYIG....EPIDMIGINYYSMSVN   310
Bp.txt         ..........IQDGDMDIIG....EPIDMIGINYYSMSVN   300
Bs.txt         MKNENQLLPLAMKGRIALIGPMADAANNMCGMWSPTCDPS   437
Pa.txt         ..........IQDGDMDIIG....EPIDMIGINYYSMSVN   300
Consensus                   g    ig        m g

502.seq        RFNPVAPFLQSEE...............INMPLPLTDIGW   335
Bp.txt         RFNPEAGFLQSEE...............INMGLPVTDIGW   325
Bs.txt         RHSSLLERIRAATKGKAEILYAKGSNVYYDAGMEAGAVGG   477
Pa.txt         RFNPEAGFLQSEE...............INMGLPVTDIGW   325
Consensus      r       l                             g

502.seq        PLVARAVYEVLHYLQKYGNIDIYFTENGACYNHEVVKRAK   375
Bp.txt         PVESRGLYEVLHYLQKYGNIDIYITENGACINDEVVN.GK   364
Bs.txt         RTLQRGDNQKLYAEAMAVASRADVIVAAVGECAEMTGESA   517
Pa.txt         PVESRGLYEVLHYLQKYGNIDIYITENGACINDEVVN.GK   364
Consensus          r     l                      e

502.seq        VQVDRRISKLQQHFQLVQRTIHDRLHVKGYMACSLLDNFL   415
[illegible]
```

Fig. 1

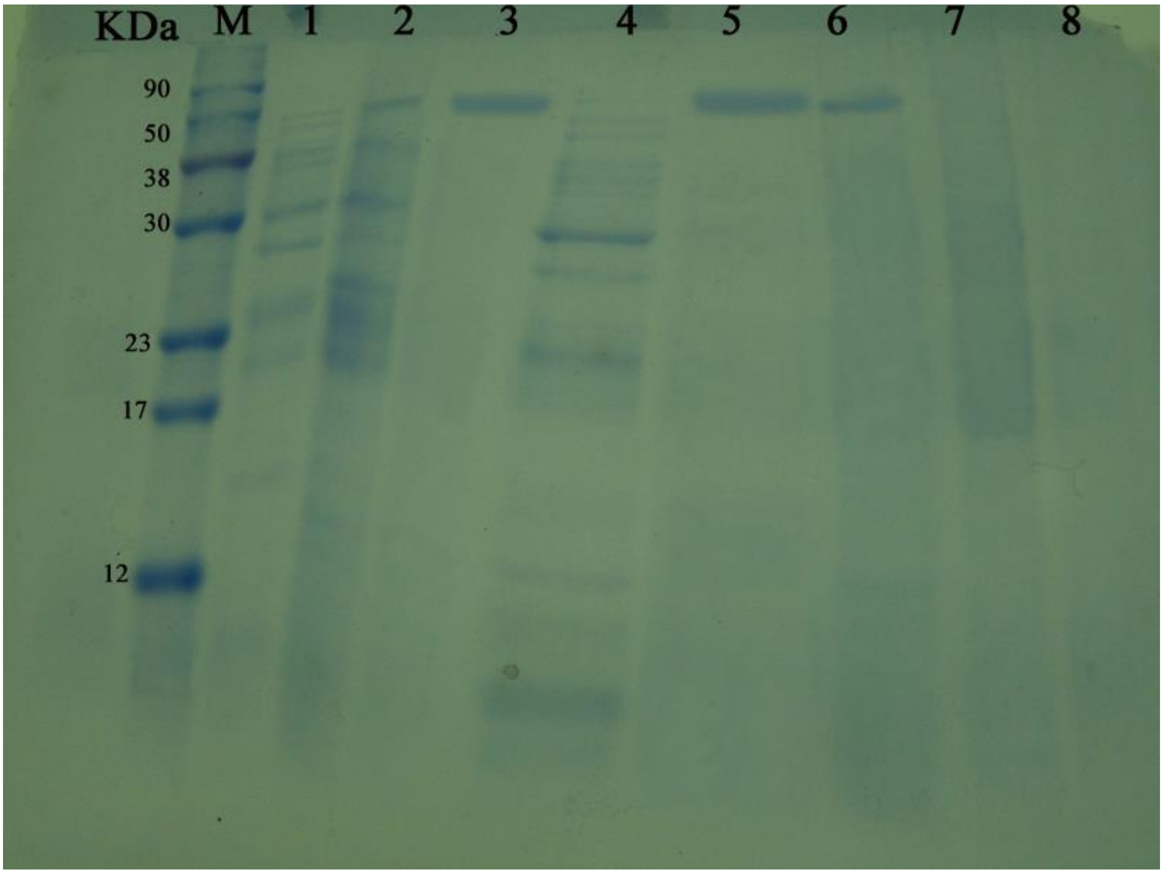

Fig. 2
